# Lactic acid fermentation as a tool to enhance the antioxidant properties of *Myrtus communis* berries

**DOI:** 10.1186/s12934-015-0250-4

**Published:** 2015-05-07

**Authors:** José Antonio Curiel, Daniela Pinto, Barbara Marzani, Pasquale Filannino, Giovanni Antonio Farris, Marco Gobbetti, Carlo Giuseppe Rizzello

**Affiliations:** Dipartimento di Scienze del Suolo, della Pianta e degli Alimenti, University of Bari, Via G. Amendola 165/a, Bari, 70126 Italy; Giuliani S.p.A., Milan, 20129 Italy; Dipartimento di Scienze ambientali agrarie e biotecnologie agro-alimentari, Università degli Studi di Sassari, Sassari, Italy

**Keywords:** Myrtle, Fermentation, Lactic acid bacteria, Antioxidant activity

## Abstract

**Background:**

Myrtle (*Myrtus communis* L.) is a medicinal and aromatic plant belonging to Myrtaceae family, which is largely diffused in the Mediterranean areas and mainly cultivated in Tunisia and Italy. To the best of our knowledge, no studies have already considered the use of the lactic acid fermentation to enhance the functional features of *M. communis*. This study aimed at using a selected lactic acid bacterium for increasing the antioxidant features of myrtle berries, with the perspective of producing a functional ingredient, dietary supplement or pharmaceutical preparation. The antioxidant activity was preliminarily evaluated through *in vitro* assays, further confirmed through *ex vivo* analysis on murine fibroblasts, and the profile of phenol compounds was characterized.

**Results:**

Myrtle berries homogenate, containing yeast extract (0.4%, wt/vol), was fermented with *Lactobacillus plantarum* C2, previously selected from plant matrix. Chemically acidified homogenate, without bacterial inoculum and incubated under the same conditions, was used as the control. Compared to the control, fermented myrtle homogenate exhibited a marked antioxidant activity *in vitro*. The radical scavenging activity towards DPPH increased by 30%, and the inhibition of linoleic acid peroxidation was twice. The increased antioxidant activity was confirmed using Balb 3 T3 mouse fibroblasts, after inducing oxidative stress, and determining cell viability and radical scavenging activity through MTT and DCFH-DA assays, respectively. The lactic acid fermentation allowed increased concentrations of total phenols, flavonoids and anthocyanins, which were 5–10 times higher than those found for the non-fermented and chemically acidified control. As shown by HPLC analysis, the main increases were found for gallic and ellagic acids, and flavonols (myricetin and quercetin). The release of these antioxidant compounds would be strictly related to the esterase activities of *L. plantarum*.

**Conclusions:**

The lactic acid fermentation of myrtle berries is a suitable tool for novel applications as functional food dietary supplements or pharmaceutical preparations.

## Background

Myrtle (*Myrtus communis* L.) is a medicinal and aromatic plant belonging to Myrtaceae family, which is largely diffused in the Mediterranean areas and mainly cultivated in Tunisia and Italy [1]. Nowadays, leaves and berries are largely used as spice, in food processing [2] and cosmetic industry [3]. In Italy, especially in Sardinia, berries and leaves are used for the manufacture of two celebrated liquors, named *Mirto rosso* and *Mirto bianco* [1]. In traditional medicine, myrtle is frequently consumed as infusion and decoction [4]. The infusion of leaves is considered stimulant, antiseptic, astringent and hypoglycemic. It is also used to treate eczema, psoriasis, asthma, gastrointestinal disorders, urinary infections and diarrhea [5]. The leaf decoction is still used for vaginal washing, enemas and against respiratory diseases [6]. The decoction from fruits is used as antidiarrheal, antihemorrhoidal agents, and to treat mouth and eyes diseases [5]. Traditionally, flowers are used for the treatment of varicose veins [4].

Despite the well-established use of myrtle in traditional medicine and the increasing scientific interest, there is a lack of summarized data on the functional compounds coming from the different preparations, therapeutical applications, and the possible risks regarding the use [7].

The different parts of the *Myrtus communis* plant were chemically characterized, and several bioactive compounds were found at various levels. Leaf and flowers are rich in essential oils, tannins, phenolic acids and flavonoids [8,9]. Fruits are rich of volatile compounds, tannins, sugars, anthocyanins, fatty acids and organic acids such as citric and malic acids [2]. Several studies have focused the antioxidant, antimicrobial and anticancer features of various myrtle extracts [1,10], with emphasis on essential oils and organic solvent extracts. Recently, the phytochemical composition and biological activities of a myrtle infusion [1], the most common preparation for using berries and leaves, were also investigated. A potential dietary source for health-protective compounds was claimed [1].

Based on the above literature, two main priorities would emerge. First, the myrtle potential antioxidant activity should be better defined and, possibly, enhanced. Second, standardized and novel formulations for traditional or innovative commercial applications should be exploited [11].

Lactic acid bacteria and, more in general, the lactic acid fermentation is considered as one of the most suitable tool to exploit the biogenic/functional potential of plant matrices and to enrich them with bioactive compounds [12]. Indeed, the fermentation by selected lactic acid bacteria was largely used to enhance the antimicrobial, antioxidant and immune-modulatory features of several cereal, pseudo-cereal and leguminous flours as well as of medicinal plants like *Echinacea* spp. [11].

To the best of our knowledge, no studies have already considered the use of the lactic acid fermentation to enhance the functional features of *M. communis*. This study aimed at using a selected lactic acid bacterium for increasing the antioxidant features of myrtle berries, with the perspective of producing a functional ingredient, dietary supplement or pharmaceutical preparation. The antioxidant activity was preliminarily evaluated through *in vitro* assays, further confirmed through *ex vivo* analysis on murine fibroblasts, and the profile of phenol compounds was characterized.

## Results

### Myrtle fermentation

The homogenate of myrtle berries with distilled water (pH 5.08 ± 0.21) allowed a very poor growth of *L. plantarum* C2 (initial cell density 5 × 10^7^ cfu/ml) at 30°C. The addition of glucose (1%) did not lead to significant (*P <* 0.05) variation of both the values of pH and cell density after 24 or 48 h of incubation. Contrarily, when yeast extract (0.4%, wt/vol) was added to homogenate, the cell density of the starter increased by ca. 2 logarithmic cycles after 24 h, remaining stable up to 48 h of incubation. The value of pH decreased to 4.56 ± 0.32 and 3.40 ± 0.28, respectively after 24 and 48 h of fermentation. Enterobacteria were not detectable by plate count in 1 ml of homogenate. All further experiments referred to myrtle homogenate, which was supplemented with yeast extract and fermented for 48 h (optimal culture conditions).

Under the above optimal conditions, the kinetic of acidification was characterized by values of *A* (ΔpH), latency phase duration (λ, expressed in hours), and V_max_ (∆pH/h) of 0.58 ± 0.02, 0.92 ± 0.3, and 0.09 ± 0.02, respectively. The parameters for the kinetic of growth were: A, 2.21 ± 0.05 log cfu/g; λ, 0.72 ± 0.03 h; and μ_max_, 0.32 ± 0.04 (∆log cfu/g/h).

#### *In vitro* antioxidant activity

A myrtle homogenate fermented for 48 h at 30°C with *L. plantarum* C2 (Mf) and a myrtle homogenate non-inoculated and chemically acidified, incubated under the same conditions (Mct), were assayed for the antioxidant activity *in vitro* by three different methods.

First, the antioxidant activity was assayed as radical scavenging activity on stable 2,2-diphenyl-1-picrylhydrazyl (DPPH) radical. The analysis was carried out using methanol extracts (ME). During radical scavenging assay, the colored stable DPPH radical is reduced to non-radical DPPH-H, when in the presence of an antioxidant or a hydrogen donor. DPPH radical, without antioxidants, was stable over the time. Under the assay conditions, the 100% of activity corresponded to the complete scavenging of DPPH radical (50 μM final concentration) after 10 min of incubation with the antioxidant compounds. According to previous studies [11,13], the color intensity of DPPH^•^ showed a logarithmic decline when it was in the presence of butylated hydroxytoluene (BHT). The activity of Mct was significantly (*P* < 0.05) lower than that of BHT, which was used as the positive control, and had a radical scavenging activity towards the stable radical DPPH of 76 ± 1% (Table 1). Fermentation significantly (*P* < 0.05) increased the radical scavenging activity, it was ca. 5% higher than BHT.

**Table 1:** ***In vitro*** **antioxidant activity**

	DPPH radical scavenging activity (%)*	Lipid peroxidation inhibitory activity (%)*	**Radical cation ABTS** ^**+**^ **scavenging activity (mM Trolox equiv.)**
**BHT**	76 ± 1^b^	55 ± 2^b^	nd
**Mct**	60 ± 2^c^	52 ± 3^b^	26.6 ± 0.1^b^
**Mf**	81 ± 3^a^	84 ± 3^a^	56.6 ± 0.2^a^

Radical scavenging activity towards DPPH^•^, linoleic acid peroxidation inhibitory activity, and radical cation ABTS^+^ scavenging activity of the myrtle homogenate fermented for 48 h at 30°C with *Lactobacillus plantarum* C2 (Mf). The chemically acidified myrtle homogenate, without bacterial inoculum, and incubated for 48 h at 30°C (Mct), was the control. Butylatedhydroxytoluene (BHT) was used as antioxidant reference.

Data are the mean of three independent analyses.

Nd: not determined.

*determined on methanol extracts (ME).

^a-c^Values with different superscript letters, in the same column, differ significantly (*P* < 0.05).

The quantification of the inhibition of linoleic acid peroxidation was also used to measure the antioxidant activity. Lipid peroxidation is thought to proceed via radical mediated abstraction of hydrogen atoms from methylene carbons in polyunsaturated fatty acids [14]. The antioxidant activity of the ME from Mct did not significantly (*P >* 0.05) differed from that of the BHT that was used as the positive control (55 ± 2%). When Mf was used, the inhibition of the linoleic acid peroxidation significantly (*P* < 0.05) increased (Table 1).

The total antioxidant capacity of Mct and Mf was then determined based on the scavenging activity towards radical cation 2,2’-azino-di-[3-ethylbenzthiazoline sulphonate] (ABTS) [15]. ABTS assay is based on the formation of the ferryl myoglobin radical from metmyoglobin and hydrogen peroxide, which causes the oxidation of ABTS to ABTS˙^+^, the chromogen radical cation. In the presence of an antioxidant agent such as Trolox (water-soluble vitamin E analog), the chromogenic reaction is suppressed. The ABTS scavenging activity of the Mf was twice with respect to that found for Mct (Table 1).

### Cytotoxicity

**Figure 1:**
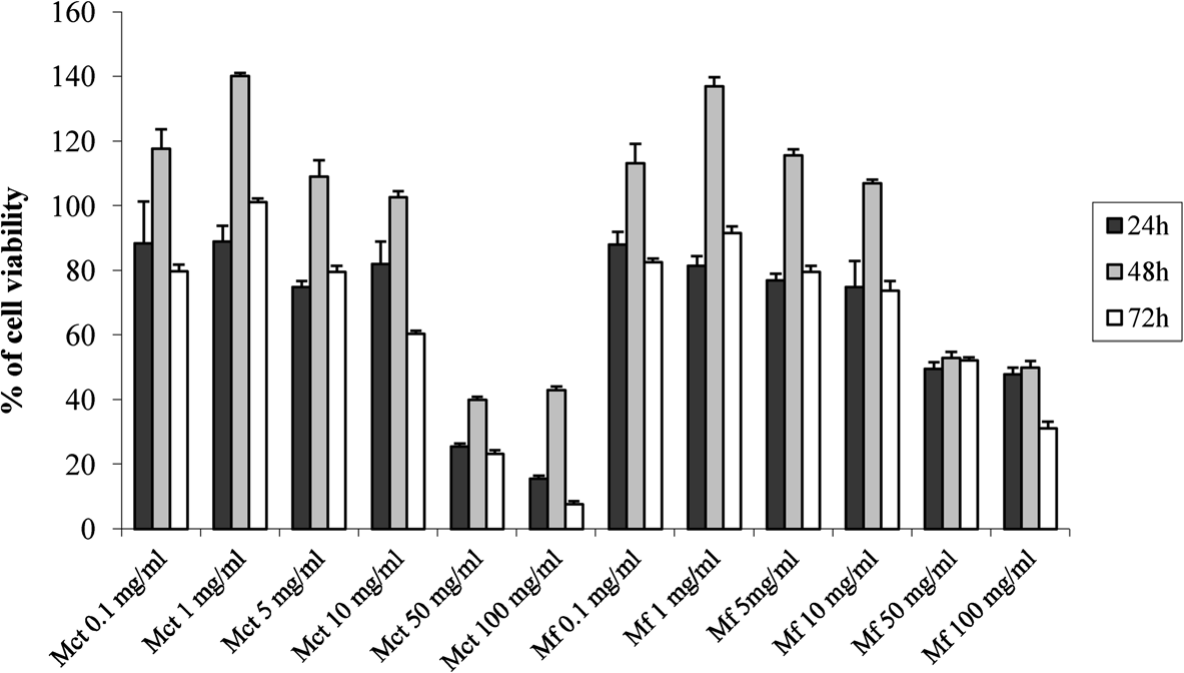
Cell viability of mouse fibroblasts. Effect of different concentrations (0.1 - 100 mg/ml) of freeze-dried myrtle berry homogenates on the cell viability of mouse fibroblasts. Mouse fibroblasts were cultured in Dulbecco’s Modified Eagle Medium (DMEM), and incubated with re-suspended freeze-dried homogenates for 24, 48, and 72 h. The percentage of viable cells was measured through the 3-(4,5-dimethylthiazol-2-yl)-2,5-diphenyltetrazolium bromide (MTT) assay. A myrtle homogenate fermented for 48 h at 30°C with *Lactobacillus plantarum* C2 (Mf) and a myrtle homogenate non-inoculated and chemically acidified, incubated under the same conditions (Mct), were assayed. α-Tocopherol (α-tp, 250, 500, and 100 μM) was used as the positive control. Data are the means of three independent experiments twice analysed.

Aiming at determining the cytotoxicity of myrtle extracts, the cell viability of the mouse fibroblasts Balb3T3 was assayed after the exposure to freeze-dried Mct and Mf. The concentrations ranged from 0.1 to 100 mg/ml. The fibroblast viability was determined through the MTT (3-(4,5-dimethyl-2-yl)-2,5-diphenyltetrazolium bromide) assay (Figure 1). Compared to the control (untreated cells in basal medium), the treatment with Mct for 24 h at concentrations higher than 50 mg/ml induced a significant (*P <* 0.05) cytotoxicity on fibroblasts. Cytotoxicity corresponded to the effect causing a decrease of the cell viability below 70%. At concentrations higher than 10 mg/ml, the cytotoxicity was manifested when the treatment was extended to 72 h. Nevertheless, a treatment lasting 48 h with Mct at concentrations lower than 10 mg/ml induced a significant (*P <* 0.05) proliferation of the cell cultures. When Mf was used (Figure 1), no cytotoxic effect was found at concentrations lower than 50 mg/ml, independently from the time of incubation. Cell proliferation was found when fibroblasts were treated for 48 h with Mf at concentrations lower than 10 mg/ml.

### Protective effect towards oxidative-induced stress in Balb3T3 cells

**Figure 2:**
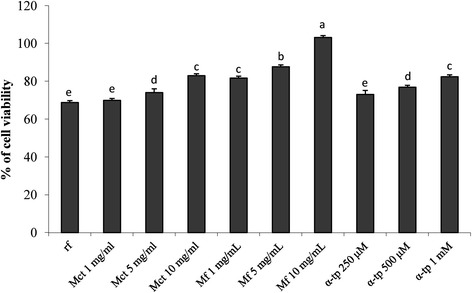
Effect on mouse fibroblasts after induced oxidative stress. Effect of different concentrations (1–10 mg/ml) of freeze-dried myrtle berry homogenates on the cell viability of mouse fibroblasts after oxidative stress. Mouse fibroblasts were cultured in Dulbecco’s Modified Eagle Medium (DMEM), and incubated with re-suspended freeze-dried homogenates for 16 h. Oxidative stress was artificially induced by incubating cultured cells with 400 μM hydroxide peroxide for 2 h. The percentage of viable cells was measured through the 3-(4,5-dimethylthiazol-2-yl)-2,5-diphenyltetrazolium bromide (MTT) assay. The viability of H_2_O_2_-stressed cells incubated without antioxidant compounds (reference, rf) was also included. A myrtle homogenate fermented for 48 h at 30°C with *Lactobacillus plantarum* C2 (Mf) and a myrtle homogenate non-inoculated and chemically acidified, incubated under the same conditions (Mct), were assayed at concentration ranging from 1 to 10 mg/ml. α-Tocopherol (α-tp; 250, 500, and 1000 μM) was used as the positive control. rf: H_2_O_2_-stressed cells. Data are the means of three independent experiments twice analysed. ^a-e^Columns with different superscript letters differ significantly (*P* < 0.05).

To further investigate the capacity of myrtle extracts to act as radical scavenger, Balb 3 T3 cells were grown in the presence of freeze-dried Mct and Mf. Afterwards, cells were treated with hydrogen peroxide. Concentrations higher than 10 mg/ml were not assayed to avoid cytotoxic effects. Cell viability was assayed through the capacity of functional mitochondria to catalyze the reduction of MTT to formazan salt via mitochondrial dehydrogenases. Compared to the negative control (68.8 ± 2.1% of cell viability after oxidative stress), α-tocopherol and both the myrtle extracts significantly (*P* < 0.05) increased cell survival (Figure 2). Overall, the protective effect of Mct was significantly (*P* < 0.05) lower than that of Mf for all the concentrations assayed. In particular, Mf at concentrations of 5 and 10 mg/ml induced the highest cell viability (87.7 ± 1.2 and 103.2 ± 1.3%, respectively). The activity of Mf was also significantly (*P* < 0.05) higher than that found with α-tocopherol, which was assayed at concentrations ranging from 250 to 1000 μg/ml.

### Intracellular reactive oxygen species (ROS) generation

**Figure 3:**
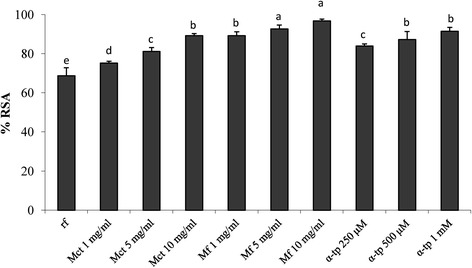
Radical scavenging activity on mouse fibroblasts. Effect of different concentrations (1–10 mg/ml) of freeze-dried myrtle berry homogenates on the radical scavenging activity (RSA) of mouse fibroblasts after oxidative stress, as estimated by 2’,7’-dichlorofluorescein diacetate (DCFH-DA) assay. Mouse fibroblasts were cultured in Dulbecco’s Modified Eagle Medium (DMEM), and incubated with re-suspended freeze-dried homogenates for 16 h. Oxidative stress was artificially induced by incubating cultured cells with 400 μM hydroxide peroxide for 2 h. The percentage of viable cells was measured through the 3-(4,5-dimethylthiazol-2-yl)-2,5-diphenyltetrazolium bromide (MTT) assay. The RSA of the H_2_O_2_-stressed cells incubated without antioxidant compounds (reference, rf) was also included. A myrtle homogenate fermented for 48 h at 30°C with *Lactobacillus plantarum* C2 (Mf) and a myrtle homogenate non-inoculated and chemically acidified, incubated under the same conditions (Mct), were assayed at concentration ranging from 1 to 10 mg/ml. α-Tocopherol (α-tp; 250, 500, and 1000 μM) was used as the positive control. rf: H_2_O_2_-stressed cells. Data are the means of three independent experiments twice analysed.^a-e^Columns with different superscript letters differ significantly (*P* < 0.05).

To determine the potential of myrtle extract against oxidative stress-mediated injuries, Balb3T3 cells were pre-treated with Mct, Mf or α-tocopherol, further incubated with 2’,7’-dichlorofluorescein diacetate (DCFH-DA), and stressed with H_2_O_2_. DCFH-DA was used as a probe to assess the formation of intracellular hydrogen peroxide by flow cytometry. The assay correlates to the emitted fluorescence with the ROS intracellular production and, consequently, to the antioxidant activity of the samples [16]. Data are reported as percentage of radical scavenging activity (RSA) with respect to the control (H_2_O_2_ stressed cells). As expected, cells pre-treated with α-tocopherol showed RSA higher than 80% (Figure 3). Similar values were found when Mct was used at concentrations ranging from 5 to 10 mg/ml. The highest activity was found for Mf. When the pre-treatment was carried out with 5 or 10 mg/ml of Mf, RSA higher than 92% and significantly (*P* < 0.05) higher than those found for Mct and 0.5-1 mM α-tocopherol were observed.

### Phenolic compounds composition

Myrtle extracts were analyzed for the concentration of total phenols. A markedly significant (*P* < 0.05) increase was found when *L. plantarum* C2 was used as starter for fermentation. Indeed, Mf contained an amount of total phenols ca. 5-times higher than that of Mct (Table 2).

**Table 2:** **Phenolic compouds profile**

**Phenolic compounds**	**Mct**	**Mf**
*Total phenols* (GAE/g dm*)	135.49 ± 2.35^b^	669.38 ± 4.19^a^
*Total flavonoids* (RE/g dm)	20.39 ± 3.40^b^	113.30 ± 2.6^a^
*Total anthocyanins* (TAC/g dm)	4.98 ± 0.63^b^	52.75 ± 1.33^a^
*Phenolic acids*		
Gallic acid (mg/g dm)	0.17 ± 0.03^b^	0.55 ± 0.02^a^
Vanillic acid (mg/g dm)	0.10 ± 0.02^b^	0.28 ± 0.02^a^
Syringic acid (mg/g dm)	0.14 ± 0.04^b^	0.28 ± 0.02^a^
Ellagic acid (mg/g dm)	1.44 ± 0.03^b^	2.78 ± 0.04^a^
*Flavonols/Flavanols*		
Myricetin (mg/g dm)	1.11 ± 0.02^b^	2.56 ± 0.03^a^
Quercetin (mg/g dm)	0.20 ± 0.01^b^	0.79 ± 0.02^a^
Catechin (mg/g dm)	1.12 ± 0.02	1.26 ± 0.03

Phenolic compounds of the myrtle homogenate fermented for 48 h at 30°C with *Lactobacillus plantarum* C2 (Mf). The chemically acidified myrtle homogenate, without bacterial inoculum, and incubated for 48 h at 30°C (Mct), was the control.

*dm: dry matter;

GAE: gallic acid equivalents.

RE: rutin equivalents.

TAC: total anthocyanins content.

^a-b^Values with different superscript letters, in the same row, differ significantly (*P* < 0.05).

Lactic acid fermentation also positively affected the concentration of total flavonoids and total anthocyanins. These compounds were, respectively, found at levels ca. 5- and 10-times higher than those of Mf (Table 2).

Free phenolic acids and flavonols of Mf were determined by HPLC and their concentrations were compared to those that were present in Mct. The highest concentrations were found for ellagic and gallic acids, which were ca. 2- and 3-times higher than those of Mct (Table 2). Also the concentrations of vanillic, and syringic acids were the highest in Mf. Flavonols (myricetin and quercetin) increased by ca. 2- and 4-times in Mf, reaching levels of 2.56 ± 0.03 and 0.79 ± 0.02 mg/g of dm, respectively (Table 2). The flavanol catechin did not significantly (*P >* 0.05) increase during fermentation.

## Discussion

Health benefits from fermentation, also in the case of plant materials, are usually direct, through interaction of ingested live microorganisms with the host (probiotic effect), or indirect as the result of the ingestion of microbial metabolites, which are synthesized during fermentation (biogenic effect) [17]. Under optimal processing conditions, microbes may contribute to plant functionality through their enzyme portfolio, which promotes the synthesis of various metabolites and/or the release of functional compounds that are cryptic in the raw matrix [17]. Recently, lactic acid bacteria were used to synthesize γ-amino butyric acid (GABA) from grape must [18], isoflavone, aglycones and equol from soy milk, antioxidant and anti-hypertensive peptides, and lunasin from various cereal and pseudo-cereal flours [19,20,21].

A large number of plants were screened to be sources of novel phenolic compounds for alimentary, cosmetic and pharmaceutical uses [22]. First, this study reported the capacity of a lactic acid bacterium to enhance the antioxidant properties and the phenol profile of myrtle berries. A selected strain of *Lactobacillus plantarum*, which was demonstrated to well adapt to plant matrices rich of polyphenols [13,19,23,24], was used. The concentration of fermentable carbohydrates of myrtle berries was enough to allow bacterial growth, but the addition of yeast extract to the homogenate was needed to get the optimal growth of *L. plantarum* C2.

Oxidative stress and lipid peroxidation are believed to play a significant role in the development of tissue damage and in several pathologies of the human body [22]. Antioxidant activities of fermented myrtle berries were investigated, and compared to a non-inoculated and chemically acidified control. Chemical acidification was carried out to exclude the effect of the pH on the antioxidant activity and phenol extractability from myrtle homogenate. The concentration of phenols largely varies among the different parts of the same plant (leaf, roots, berries), and is affected by genetic, environmental (e.g., light, temperature, agronomic practices), and processing factors (drying temperature, extraction methods, formulation and storage conditions) [25,26]. Myrtle is a rich source of phenols, and hydrolysable tannins and flavonoids are present in seed and pericarp, respectively [27]. The antioxidant activity of a plant extract is not usually related to a single phenolic compound [22]. Several *in vitro* studies indicated that flavonoids, coumarines, phenolic acid, lignans, hydroxycinnamates and stilbenes have altogether antioxidant activity [22]. The antioxidant capacity of phenols is mainly due to their redox properties, which allow them to act as reducing agents, hydrogen donors, and singlet oxygen quenchers [22].

The antioxidant activity of myrtle homogenate was first estimated *in vitro*. The radical scavenging activity of the extract fermented with *L. plantarum* C2 was markedly higher than that of the non-fermented homogenate, and it reached almost the same value of the synthetic antioxidant. A marked inhibition of the linoleic acid peroxidation as well as a relevant total antioxidant capacity (estimated towards ABTS radical) were also found, confirming the positive effect of the lactic fermentation.

The effect of phenols on cell proliferation and cytotoxicity is largely investigated and debated, aiming at explaining the potential protective role towards oxidative stresses and tumor development [28]. The MTT assay on mouse fibroblasts Balb3T3 was used to determine the cytotoxicity of the myrtle extracts. As expected, a cytotoxic effect was found for high concentrations (>50 mg/ml) of fermented and freeze-dried myrtle extract. A proliferative effect occurred at lower concentrations, with treatment lasting 48 h. The MTT assay on mouse fibroblasts was also used to show the protective effect of the fermented myrtle extract towards induced oxidative stress. The antioxidant effect on cultured cells was higher than that of the non-fermented homogenate and also higher than that of α-tocopherol, which was used at concentrations up to 1 mg/ml. The protective effect was investigated through the determination of the intracellular ROS production and detoxification by DCFH-DA assay. Also in this case, it was confirmed the markedly higher antioxidant activity on mouse fibroblasts compared to the non-fermented myrtle extract. Clearly, the antioxidant potential of myrtle berries has also to be partially attributed to the inherent phenols contained in the raw matrix [1]. Nevertheless, all the above results showed that fermentation by *L. plantarum* C2 caused a modification of the phenol profile of marked significance.

Aiming at explaining the increase of the antioxidant activity due to lactic acid fermentation, the phenolic fraction of myrtle extracts was characterized. The fermentation caused an increase of the concentration of total phenols, which was ca. 5-times higher than that found in the non-fermented homogenate. As expected, the same trend was found for flavonoids and anthocianins. These latter compounds, which are responsible for the dark blue color of myrtle berries and extracts [27] increased by ca. 10 times. The lactic acidification cannot be considered as the main cause for the above increase, since also the non-fermented homogenate was chemically acidified. The increase of the solubility of phenols due to biological acidification was already reported for many plant matrices [25,26]. The capacity of *L. plantarum* to degrade polyphenolic compounds was already documented and considered as a metabolic strategy for adapting to environmental hostile niches [29,30]. All phenolic acids increased, but mainly gallic and ellagic acids. Both these compounds may be released via tannase activity. Tannase or tannin acyl hydrolase (EC 3.1.1.20) catalyzes the hydrolysis of ester bonds that are present in hydrolysable tannins and gallic acid esters. First, Osawa et al. [31] showed the presence of tannase activity in *L. plantarum* isolates. Later, this capacity was confirmed in other strains, which were isolated from various food matrices [32,33,34]. The route to degrade tannins by *L. plantarum* implies that tannic acid is hydrolyzed to gallic acid and glucose, and the gallic acid formed is further decarboxylated to pyrogallol [35]. This pathway implies the presence of tannase and gallate decarboxylases, whose presence was previously documented in *L. plantarum* [31]. Although the metabolism of *L. plantarum* towards phenolic compounds is not completely elucidated [36], it may be hypothesized that the increase of the other phenolic acids (vanillic and syringic acids) and flavonols (myricetin and quercetin) depends on feruloyl esterases that are active on complex phenolic molecules or glycosylated forms. These latter are very abundant in myrtle fruits [1,37]. It was observed that flavol aglycones (e.g., myricetin and quercetin) contain multiple OH groups and show higher antioxidant activity than their glycosides (e.g., rutin and myricitrin) [2]. Feruloyl esterases are widespread in *L. plantarum* strains, and allow to metabolize compounds that are abundant in fermented plant matrices (e.g., hydroxycinnamoyl esters) [36]. Similar effects by lactic acid bacteria on plant phenols were documented for cowpeas [38], onions [39], pomegranate [40], and wheat flour [26]. Catechin, a flavanol largely present in many fruits [41], was not affected by lactic acid fermentation. Contrarily to other classes of flavonoids, flavanols are not glycosylated in plant matrices [42], thus excluding a role of the esterase activity.

## Conclusions

Nutraceutical industry and preventive medicine are currently showing a marked interest for natural antioxidant compounds because of their potential application in food, cosmetic and pharmaceutical products to replace synthetic carcinogenous antioxidants [7]. The demand for dietary phytonutrients encourages the exploitation of plant potential through lactic acid fermentation [12]. In agreement with this perspective, this study demonstrates how the antioxidant properties of myrtle berries could be enhanced through lactic acid fermentation. Depending on the application, the fermented myrtle berry homogenate may be used as such or, separated from the solid phase, as supernatant (in aqueous or freeze-dried forms). Although *in vivo* assay should be further carried out, novel applications as functional food dietary supplements or pharmaceutical preparations of the fermented myrtle berries should be warranted.

## Methods

### Microorganisms and culture conditions

*Lactobacillus plantarum* C2, which was previously isolated from carrots, identified by partial sequencing of 16S rRNA, and selected for vegetable fermentations [11,23,24,42,43], was used in this study. The strain belongs to the Culture Collection of the Department of Soil, Plant, and Food Sciences (Bari, Italy). *L. plantarum* C2 was cultivated at 30°C onto MRS broth (Oxoid, Basingstoke, Hampshire, United Kingdom). When used for fermentation, lactic acid bacteria cells were cultivated until the late exponential phase of growth was reached (ca. 10 h), washed twice in 50 mM phosphate buffer, pH 7.0, and re-suspended in the liquid substrate. Enumeration of lactic acid bacteria was carried out by plating onto MRS agar at 30°C for 48 h. Total enterobacteria were determined on Violet Red Bile Glucose Agar (VRBGA, Oxoid) at 37°C for 24 h.

### Fermentation

Ripe berries were collected manually (autumn 2013) from spontaneous *Myrtus communis* plants harvested in Sardinia, Italy, and stored at −20°C until they were used as substrate for fermentation. The substrate was obtained as reported by Liu et al. [44], with some modifications. In details, myrtle berries were added to distilled water (1:1, wt/wt) and homogenized using a Stomacher 400 lab blender (Seward Medical, London) for 10 min. The homogenate was kept at 4°C for 2 h. Then, the homogenate was inoculated with the starter, without any supplementation (i), or with the addition of 0.4% (wt/vol) yeast extract (Oxoid) (ii), or 1% glucose (wt/vol) (iii). The initial cell density of the lactic acid bacterium was of ca. 5 × 10^7^ cfu/ml. Fermentation (Mf) was allowed at 30°C for 48 h, under stirring conditions (120 rpm). An homogenate without bacterial inoculum, and chemically acidified with lactic acid (final pH 4.0), was incubated under the same conditions and used as the control (Mct). Mct and Mf were centrifuged (10,000 *× g* for 20 min) at 4°C, and the supernatants were used for analyses.

### Kinetics of growth and acidification

Kinetics of growth and acidification were determined and modelled in agreement with the Gompertz equation, as modified by Zwietering et al. [45]: *y* = k + A exp{− exp[(μmax or Vmax e/A)(λ-t) + 1]}; where *y* is the growth expressed as log cfu/g or the acidification rate expressed as dpH/dt (units of pH) at the time t; k is the initial level of the dependent variable to be modelled (log cfu/g or pH units); A is the cell density or pH (units) variation (between inoculation and the stationary phase); *μ*_max_ or V_max_ is the maximum growth rate expressed as *Δ*log cfu/g/h or the maximum acidification rate expressed as dpH/h, respectively; *λ* is the length of the lag phase measured in hours. The experimental data were modelled by the non-linear regression procedure of the Statistica 8.0 software (Statsoft, Tulsa, USA).

### Antioxidant activity *in vitro*

First, the antioxidant activity of myrtle homogenates was evaluated *in vitro* using different methods.

The free radical scavenging capacity was determined on methanol extract (ME) using the stable 2,2-diphenyl-1-picrylhydrazyl radical (DPPH˙). ME were prepared resuspending 1 mg of freeze-dried Mct or Mf in 1 ml of 80% methanol. The protocol previously reported by Yu et al. [46] was used [11]. The reaction was monitored by reading the absorbance at 517 nm every 2 min for 30 min. A blank reagent was used to verify the stability of DPPH˙ over the test time. The absorbance value measured after 10 min was used for the calculation of the μmoles DPPH˙ scavenged by extracts. Butylated hydroxytoluene (BHT, 75 ppm) was used as the antioxidant reference [11,13,25].

The antioxidant activity of ME was also measured according to the method of Osawa and Namiki [47], with some modification [11]. One ml of ME was added to 1 ml of linoleic acid (50 mM), previously dissolved in ethanol (99.5%). Incubation in glass test tube, tightly sealed with silicone rubber cap, was allowed at 60°C in the dark for 8 days. The degree of oxidation was determined by measuring the value of ferric thiocyanate, according to Mitsuta et al. [48]. One hundred microliters of the above sample were mixed with 4.7 ml of 75% (v/v) ethanol, 0.1 ml of 30% (w/v) ammonium thiocyanate and 0.1 ml of 0.02 M ferrous chloride, dissolved in 1 M HCl. After 3 min, the color development (degree of linoleic acid oxidation) was measured spectrophotometrically at 500 nm. BHT (1 mg/ml) was used as the antioxidant references. A negative control (without antioxidant) was also considered. The inhibition effect was expressed as follows: inhibition of linoleic acid autoxidation (%) = [(negative control absorbance – sample absorbance)/negative control absorbance] × 100.

Finally, the radical cation (2,2’-azino-di-[3-ethylbenzthiazoline sulphonate]) (ABTS˙^+^) scavenging capacity of Mct and Mf was measured using the Antioxidant Assay Kit CS0790 (Sigma Chemical Co.), following the manufacturer’s instruction. Trolox (6-hydroxy 2,5,7,8-tetramethylchroman-2-carboxylic acid) was used as the antioxidant standard. The scavenging activity of was expressed as trolox equivalent (mmol/l of myrtle homogenate).

### Concentration of total phenols, flavonoids and anthocyanins

Total phenols, flavonoids and anthocyanins were determined on the ME obtained from Mct and Mf. In details, aliquots of myrtle homogenates were centrifuged to remove the solid phase (10000 × *g* for 20 min); then, the supernatants were freeze-dried and resuspended (1:1) in 80% methanol. The ME were purged with nitrogen stream for 30 min, under stirring condition, and centrifuged at 4,600 × *g* for 20 min. ME were transferred into test tubes, purged with nitrogen stream and stored at ca. 4°C before analysis. The concentration of total phenols was determined as described by Slinkard and Singleton [49]. It was expressed as gallic acid equivalent.

The concentration of flavonoids was estimated according to the aluminum chloride colorimetric method of Djeridane et al. [50]. Briefly, 1 ml of ME was mixed with 1 ml of 2% AlCl_3_ methanolic solution. After incubation at room temperature for 15 min, the absorbance was measured at 430 nm. Flavonoid concentration was calculated from a calibration curve obtained with rutin (Sigma Aldrich CO., St. Louis, MO) and expressed as milligrams of rutin equivalent/gram of dry matter (mg RE/ g dm). Data were calculated as the mean of the results obtained in three different experiments.

The concentration of total anthocyanins (TAC) was measured by a pH-differential method proposed by Ozgen et al. [51], using two buffer systems: potassium chloride buffer, pH 1.0 (0.0025 M) and sodium acetate buffer, pH 4.5 (0.4 M). Briefly, 1 ml of ME was mixed with 4 ml of each buffer and read at 510 and 700 nm against distilled water as a blank. Absorbance was calculated as A = (A_510_ – A_700_) pH 1.0 – (A_510_ – A_700_) pH 4.5. TAC of samples (mg cyanidin-3glucoside/l of extract) was calculated using the following equation: TAC (mg/l) = *A × MW × DF ×* 10^3^/ε *× L* (where A is the absorbance; MW is the molecular weight of cyanidin-3-glucoside, 449.2 Da; DF is the dilution factor; *ε* is the cyanidin-3-glucoside molar absorbance, 26,900; L is the cell path length, 1 cm) and expressed on dm.

### Extraction and analysis of phenolic compounds

Extraction of phenolic compounds was carried out following the procedure of Svensson et al. [52], with some modifications. Fifty milliliters of myrtle extracts (Mct and Mf) were mixed with 200 ml of 70% (vol/vol) methanol. The mixture was shaken for 1 h (room temperature) and then centrifuged at 4225 × *g* for 10 min. The supernatant was collected, and the pellet was subjected to a second extraction cycle. Methanol was evaporated under vacuum at 35°C using a Speed-Vac centrifuge at 35°c (Thermo Scientific, Waltham, MA). Dry matter was re-dissolved in Milli-Q water (50 ml) and acidified to pH 1.5 with hydrochloric acid. Ethyl acetate (200 ml, Sigma-Aldrich) was added, and the samples were shaken every 10 min for 30 min at room temperature. The liquid-liquid extraction was repeated two times, and ethyl acetate evaporated under vacuum at 35°C. Dry matter was re-dissolved in 10 ml of methanol.

The extract was filtered through a 0.2 μm-pore-size polytetrafluoroethylene filter (PTFE) (VWR International) and analysed by High Pressure Liquid Chromatography (HPLC) with the method of Curiel et al. [53], with some modifications. An ÄKTA Purifier system (GE Healthcare) equipped with an XTerra reversed-phase C_18_ column (Waters) was used. Gradient elution was performed at 30°C with a flow rate of 0.8 ml/min, using a mobile phase composed of solvent A (water/acetic acid, 98:2) and solvent B (water/acetonitrile/acetic acid, 78:20:2). The solvent B concentration was increased linearly from 0 to 80% between 0 and 55 min; from 80 to 90% between 55 and 57 min; 90% isocratic between 57 and 70 min; from 90 to 95% between 70 and 80 min; from 95 to 100% between 80 and 90 min. After, washing and equilibration were carried out. UV detector was set at 280 nm. The identification of compounds was carried out by comparing the retention times and area data of each peak with those of standards from Sigma Aldritch Co.

### MTT assay and protective effect on oxidative-induced stress in Balb 3 T3 cells

Mouse fibroblasts (Balb 3 T3, clone CCL-163™) were purchased from ATCC Culture Collection (Middlesex, UK), and were cultured under humidified atmosphere (5% CO_2_, 37°C), using Dulbecco’s Modified Eagle Medium (DMEM), which was supplemented with 10% (w/v) calf bovine serum (CBS), 1% penicillin (10,000 U/mL)/streptomycin (10,000 U/mL) mixture, and 1% non-essential amino acid solution (NEAA). The culture medium was renewed every two days and after four passages the cultures were used for viability assays.

Cell viability was measured using the MTT (3-(4,5-dimethyl-2-yl)-2,5-diphenyltetrazolium bromide) method [54]. The capacity of succinate dehydrogenase to convert 3-(4,5-dimethylthiazol-2-yl)-2,5-diphenyltetrazolium bromide into visible formazan crystals was assessed. For MTT assay, cells were seeded into 96-well plate (Becton Dickinson France S.A., Meylan Cedex, France) at the density of 5 × 10^4^ cells/well and incubated for 24 h.

In order to determine the non-cytotoxic concentration, cells were treated with Mct and Mf and incubated for 24, 48, and 72 h. In particular, the concentrations of freeze-dried Mct and Mf in the reaction mixture were 0.1, 1, 5, 10, 50, and 100 mg/ml. A control in basal medium, without addition of Mct or Mf, was used. After incubation, medium was removed from each well and 100 μl of MTT (0.5 mg/ml final concentration), dissolved in DMEM, were added and incubated (37°C, 5% CO_2_) in the dark for 3 h. Finally, 100 μl of dimethyl sulphoxide (DMSO) were added to dissolve purple formazan products. The solution was shacked in the dark for 15 min at room temperature. The absorbance of the solution was read at 570 nm in a microplate reader (BioTek Instruments Inc., Bad Friedrichshall, Germany).

MTT assay was also used for the determination of the viability of H_2_O_2_-stressed Balb 3 T3 cells [11,13], In this case, cells were incubated with Mct and Mf for 16 h. The concentrations of freeze-dried Mct and Mf in the reaction mixture were 1, 5, and 10 mg/ml. DMEM medium with 2.5% CBS, 1% penicillin (10,000 U7ml)/streptomycin (10,000 U/ml) and 1% non-essemtial amino acids solution (NEAA) was used as sunstrate. A negative control, without addition of Mct or Mf, was used. α-Tocopherol (250, 500 and 1000 μg/ml) was used as the positive control. After treatment, medium was removed from each well and, after washing, cells were exposed to 400 μM hydrogen peroxide (100 μl/well) for 2 h. Two controls, one without addition of Mct or Mf, and another without hydrogen peroxide treatment, were used.

After the incubation, MTT assay was performed as described above. Data were expressed as the mean percentage of viable cells compared to the control culture, without oxidative stress.

Each experiment was carried out in triplicate.

### Intracellular reactive oxygen species (ROS) generation

Production of reactive oxygen species (ROS) was monitored spectrofluorometrically on mouse fibroblasts Balb 3 T3 using the 2’,7’-dichlorofluorescein diacetate (DCFH-DA) assay, as described by Tobi et al. [16].

In details, cells were cultivated and treated for 16 h with 1, 5, and 10 mg/ml of Mct and Mf as described above. H_2_O_2_-stressed cells were used as control; DMEM (2.5% CBS) was used as culture medium. After the incubation with Mct and Mf, the medium was removed and washed twice with phosphate buffer saline (PBS). Cells were treated with DCFH-DA dissolved in DMSO (final concentration 100 μM) for 30 min at 37°C, in the dark.

Cells were then treated for 2 h at 37°C in the dark with 100 μl of pre-warmed DMEM (2,5% CBS) containing 400 μM H_2_O_2_. At the end of the treatment, cells were washed twice, lysed with Cell Lytic M lysis buffer (Sigma Aldrich), added with 1% protease inhibitor cocktail (Sigma Aldritch) and transferred into a black 96-well plate. Fluorescent 2’,7’-dichlorofluorescein (DCF) was read fluorometrically using a Fluoroskan Ascent FL Microplate Fluorescence Reader (Thermo Scientific) at excitation and emission wavelengths of 485 and 538 nm, respectively. Each experiment was carried out in triplicate.

### Statistical Analysis

Data were subjected to one-way ANOVA; pair-comparison of treatment means was achieved by Tukey’s procedure at *P* < 0.05, using the statistical software, Statistica for Windows (Statistica7.0 per Windows). Student’s *t*-test was used for MTT assay (GraphPAD 6.0 for Windows).
